# Use of iliac crest allograft for Dega pelvic osteotomy in patients with cerebral palsy

**DOI:** 10.1186/s12891-018-2293-2

**Published:** 2018-10-16

**Authors:** Ki Hyuk Sung, Soon-Sun Kwon, Chin Youb Chung, Kyoung Min Lee, Jaeyoung Kim, Moon Seok Park

**Affiliations:** 1 Department of Orthopaedic Surgery, Seoul National University Bundang Hospital, 82 Gumi-ro 173 Beon-gil, Bundang-Gu, Sungnam, Gyeonggi, 13620 South Korea; 2 Department of Mathematics, College of Natural Sciences, Ajou University, Suwon, Gyeonggi, South Korea; 3 Department of Orthopaedic Surgery, H Plus Yangji Hospital, Seoul, South Korea

**Keywords:** Dega osteotomy, Iliac crest allograft, Cerebral palsy, Goldberg score, Aceteabular dysplasia

## Abstract

**Background:**

Dega pelvic osteotomy is commonly performed procedure in patients with cerebral palsy (CP) undergoing hip reconstructive surgery for hip displacement. However, there has been no study investigating the outcomes after Dega pelvic osteotomy using allograft in patients with CP. This study investigated the outcomes of Dega pelvic osteotomy using iliac crest allograft in CP with hip displacement and the factors affecting allograft incorporation.

**Methods:**

This study included 110 patients (150 hips; mean age 8y7mo; 68 males, 42 females) who underwent hip reconstructive surgeries including Dega pelvic osteotomy using iliac crest allograft. To evaluate the time of allograft incorporation, Goldberg score was evaluated according to the follow-up period on all postoperative hip radiographs. The acetabular index, migration percentage, and neck-shaft angle were also measured on the preoperative and postoperative follow-up radiographs.

**Results:**

The mean estimated time for allograft incorporation (Goldberg score ≥ 6) was 1.1 years postoperatively. All hips showed radiographic union at the final follow-up and there was no case of graft-related complications. Patients with Gross Motor Function Classification System (GMFCS) level V had 6.9 times higher risk of radiographic delayed union than those with GMFCS level III and IV. Acetabular index did not increase during the follow-up period (*p* = 0.316).

**Conclusions:**

Dega pelvic osteotomy using iliac crest allograft was effective in correcting acetabular dysplasia, without graft-related complications in patients with CP. Furthermore, the correction of acetabular dysplasia remained stable during the follow-up period.

## Background

Cerebral palsy (CP) is defined as a group of permanent motor impairment disorders that are attributed to non-progressive disturbances in the brain of a developing fetus or infant. [1] Hip displacement (subluxation or dislocation) is common deformity in CP patients with severe impairment and is associated with acetabular dysplasia. [2] It can lead to pain and severe contractures, resulting in difficulties with perineal care, sitting balance, standing, and walking, as well as reduced quality of life. [3] Severely subluxated or dislocated hip can be corrected by hip reconstructive surgeries including proximal femoral varus osteotomy (FVO), either separately or in combination with several different types of pelvic osteotomy. [4] In patients with adequate sourcil and presence of a triradiate cartilage, reconstruction of the acetabulum using the Dega technique stabilizes the pelvis better than other techniques because it is a stable and incomplete osteotomy, and does not affect the medial cortex of the ilium. [5]

Most studies reported the use of iliac crest or femoral autograft as the interposition material for Dega osteotomy. The stability and the maintenance of osteotomy are dependent on the strength of the graft materials. [6] However, patients with CP have the osteoporotic features around the hip joint. [7] When an autogenous bone graft from the iliac crest is used, it may cause growth disturbances in the iliac bone due to splitting of the iliac apophysis, longer operation time, and increased blood loss. [8, 9] Therefore, our institution has been used iliac crest allograft as an interposition material for the Dega osteotomy in patients with CP.

Tricortical iliac allograft bone is widely available, has no donor site morbidity for harvesting, and has similar bone union rates as an autograft. [10, 11] Nevertheless, an allograft poses some concerns about the risk of transmission of infectious disease and graft rejection. [12, 13] However, a bone demineralization process can decrease the rates of disease transmission. [14] Several studies have reported allograft failure after operations on the spine, humerus, tibia and calcaneus. [15–19] However, to our knowledge, no study has investigated the outcomes after Dega pelvic osteotomy using allograft in patients with CP.

In the present study, we aimed to investigate the outcomes after Dega pelvic osteotomy, using iliac crest allograft in patients with CP. Furthermore, we also investigated the factors influencing allograft incorporation.

## Methods

### Participants

The inclusion criteria were (1) consecutive children with CP with hip displacement (2) patients who underwent hip reconstructive surgeries, including Dega pelvic osteotomy and FVO from 2003 to 2015, (2) patients with a minimum follow-up of 1 year, and (3) patients who had preoperative and at least two postoperative follow-up hip radiographs. Patients with a history of hip surgery and with inappropriate hip radiographs for assessment were excluded.

### Surgical protocol

At our hospital, hip reconstructive surgeries, including Dega pelvic osteotomy and FVO, were performed in displaced hips by two pediatric orthopedic surgeons. Hip reconstructive surgery was indicated in patients with a migration percentage (MP) of more than 33%. For FVO, the osteotomy site at the intertrochanteric level was fixated using a blade plate (Stryker, Selzach, Switzerland) or a pediatric locking compression plate (Depuy Synthes, MA, USA). For Dega pelvic osteotomy, the osteotomy site was widened using a laminar spreader until sufficient coverage of the femoral head was achieved under C-arm fluoroscopy. A tricortical iliac crest allograft was trimmed and inserted into the osteotomy site. Internal fixation of the bone graft was not performed. After surgery, bilateral short leg cast with an abduction bar were applied to maintain hip abduction position for 6 weeks. [20] Thereafter, all patients returned to a local rehabilitation center to begin standing and weight-bearing exercises.

### Consensus building

**Fig. 1:**
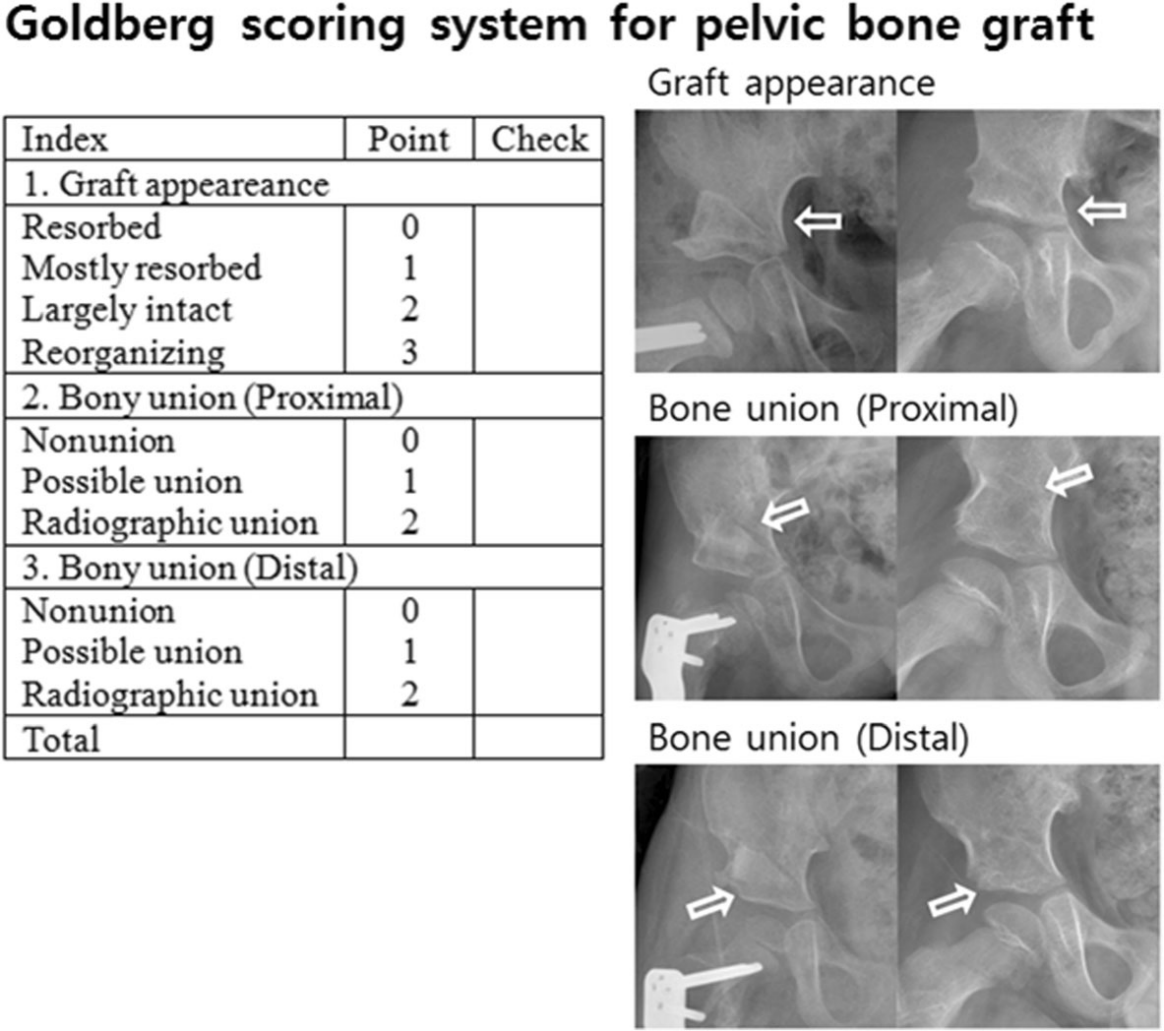
Indices of the Goldberg scoring system are shown. A postoperative hip radiograph is used for the checklist. There was no case with scores 0 and 1 for graft appearance, and no case with score 0 for proximal and distal bony union. Modified from Goldberg VM, Powell A, Shaffer JW, Zika J, Bos GD, Heiple KG. Bone grafting: role of histocompatibility in transplantation. J Orthop Res. 1985;3:389–404. Copyright © 1985 Orthopaedic Research Society

A consensus building session was conducted for the selection of the radiographic parameters; this session included 5 orthopedic surgeons. Previous studies regarding graft incorporation after bone grafting were reviewed, and the Goldberg scoring system was selected. [19, 21] In hip radiographs, graft appearance, bony union at the proximal end and bony union at the distal end, were defined and evaluated. For graft appearance, the score was 0 for resorbed, 1 for mostly resorbed, 2 for largely intact, and 3 for reorganizing. For bony union at the proximal and distal ends, the score was 0 for nonunion, 1 for possible union, and 2 for complete union. [19] The highest possible score was 7 points, which indicated excellent graft reorganization and radiographic union (Fig. 1). For our study, radiographic delayed union was defined as a Goldberg score < 6 by 6 months after the surgery.

**Fig. 2:**
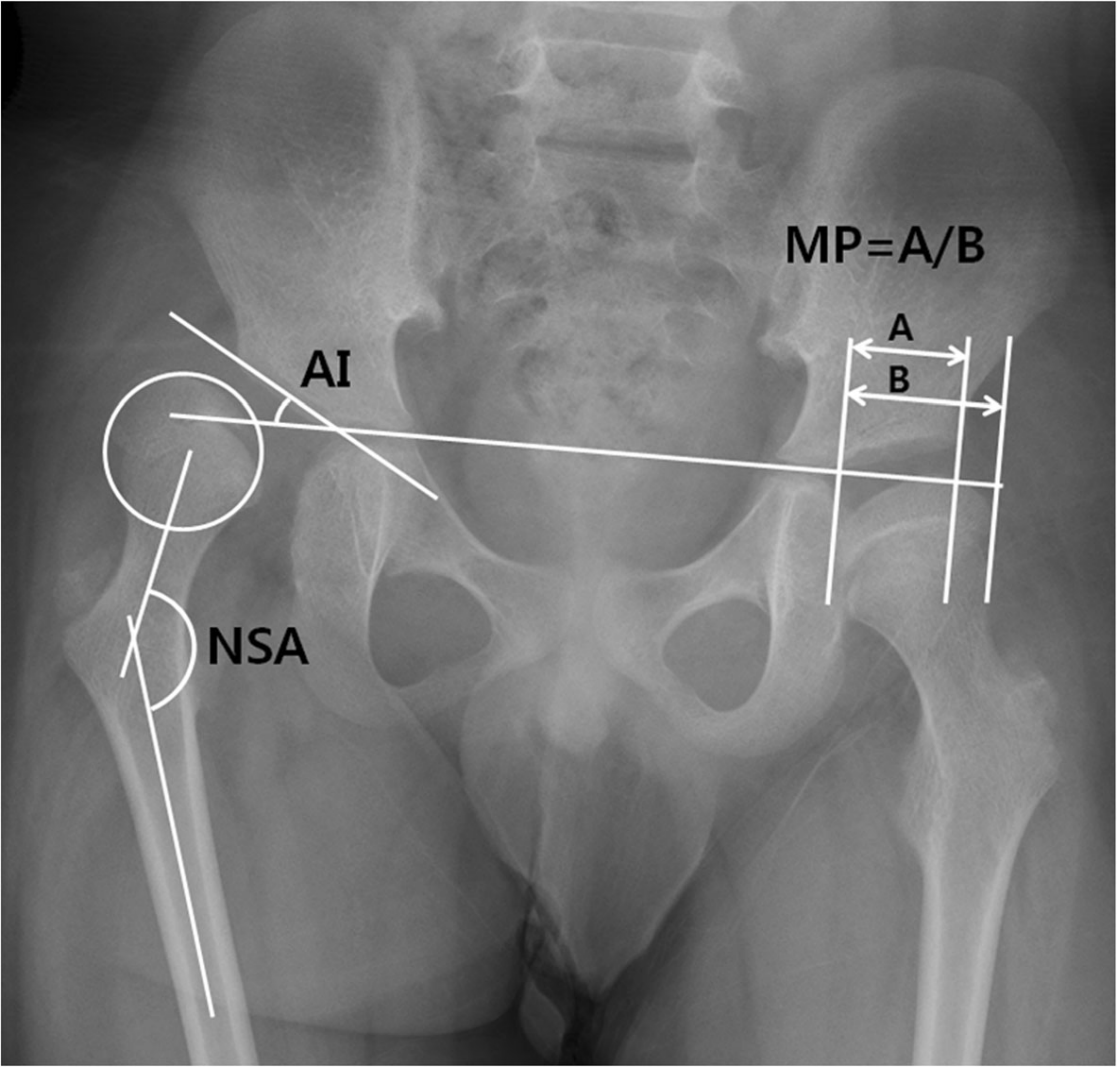
Hip internal rotation view. For the right hip neck-shaft angle (NSA) was defined as the angle between a line passing through the center of the femoral shaft and another line connecting the center of the femoral head and the midpoint of the femoral neck. The center of the femoral head was the center of the largest best-fitting circle inside the femoral head. Acetabular index (AI) was defined as the angle between the acetabular roof and the Hilgenreiner’s line. For the left hip, migration percentage (MP) was calculated by dividing the width of the femoral head lateral to Perkin’s line (**a**) by the total width of the femoral head (**b**)

Additionally, 3 radiographic parameters that were relevant to assessing hip displacement and acetabular dysplasia were selected from previous studies [3, 22–25]. These were the neck-shaft angle (NSA), MP, and acetabular index (AI) on hip radiographs (Fig. 2).

### Reliability testing and radiographic measurements

To assess the inter-observer reliabilities of radiographic measurements, three orthopedic surgeons measured the radiographic indices including MP, NSA, AI, and the Goldberg score for 36 hips independently. Four weeks after the inter-observer reliability testing, one orthopedic surgeon (JYK) performed the measurements again for 36 hips to evaluate the intra-observer reliability. After the completion of reliability test, he performed the measurement for all preoperative and postoperative follow-up hip radiographs.

### Statistical methods

Inter- and intra-observer reliabilities of radiographic measurements were assessed by the ICCs and their 95% CIs with the setting of a two-way mixed effects model, assuming a single measurement and absolute agreement. [26] Prior sample size estimation was performed for reliability testing with a target ICC value of 0.80 and a 95% CI width of 0.2 for 3 examiners. The minimum sample size was 36 hips, using Bonett’s method. [27] An ICC value more than 0.8 represented excellent reliability. Repeated measures analysis of variance with a Bonferroni post hoc test was applied to compare the preoperative radiographic measurements to postoperative and final follow-up values.

Bilateral cases were included in this study, thus, a linear mixed model (LMM) and a generalized estimating equation (GEE) were used for statistical analysis. [28] The risk factors for radiographic delayed union were evaluated by a GEE to calculate the adjusted odds ratios (ORs). The annual change in the MP, NSA, and AI was adjusted by multiple factors by using a LMM. R version 3.2.5 (R Foundation for Statistical Computing, Vienna, Austria) and SAS 9.4.2 (SAS Institute, Cary, NC, USA) were used for statistical analysis, and *p*-values less than 0.05 were considered to be significant.

## Results

One hundred ten patients with 150 hips were enrolled in this study. The mean number of follow-up radiographs was 6 per patients (range, 2–15) (Table 1).

**Table 1:** Summary of patient data

Parameters	Values
Male / Female	68 / 42
Anatomical type (diplegia / guadriplegia)	18 / 92
GMFCS level (III/I*V*/V)	17 / 39 / 54
Age at surgery (years)	8.7 ± 2.4 (2.8 to 13.8)
Follow-up duration (years)	2.9 ± 2.6 (1.0 to 12.0)
Age at final follow-up (years)	11.6 ± 3.8 (3.8 to 22.5)
Laterality (Right / Left)	80 / 70

*GMFCS* Gross Motor Function Classification System

Inter- and intra-observer reliabilities of all radiographic measurements were excellent (ICC, 0.802 to 0.924) (Table 2). MP, NSA and AI were significantly improved after hip reconstructive surgery including the Dega osteotomy (all *p* < 0.001). AI was not changed at final follow-up (*p* = 1.000), but MP and NSA had significantly increased at final follow-up (both *p* < 0.001) (Table 3).

**Table 2:** Intra- and inter-observer reliabilities of radiographic measurements

Measurements	Inter-observer reliability		Intra-observer reliability	
	ICC	95% CI	ICC	95% CI
Neck-shaft angle	0.808	0.655–0.894	0.802	0.645–0.894
Migration percentage	0.885	0.740–0.945	0.860	0.723–0.932
Acetabular index	0.817	0.709–0.895	0.833	0.732–0.904
Goldberg score	0.918	0.864–0.954	0.924	0.874–0.958

*ICC* intraclass correlation coefficient, *CI* confidence interval

**Table 3:** Summary of radiographic measurements

Radiographic index	Preoperative	Immediate postoperative	Final follow-up	*p*-value		
				Preop-postop	Preop-final	Postop-final
Acetabular index (degree)	32.2 ± 7.0	13.6 ± 5.5	13.8 ± 5.9	< 0.001	< 0.001	1.000
Neck-shaft angle (degree)	156.0 ± 9.8	119.9 ± 10.7	125.1 ± 13.6	< 0.001	< 0.001	< 0.001
Migration percentage (%)	75.2 ± 20.2	0.5 ± 2.3	11.7 ± 12.2	< 0.001	< 0.001	< 0.001

**Fig. 3:**
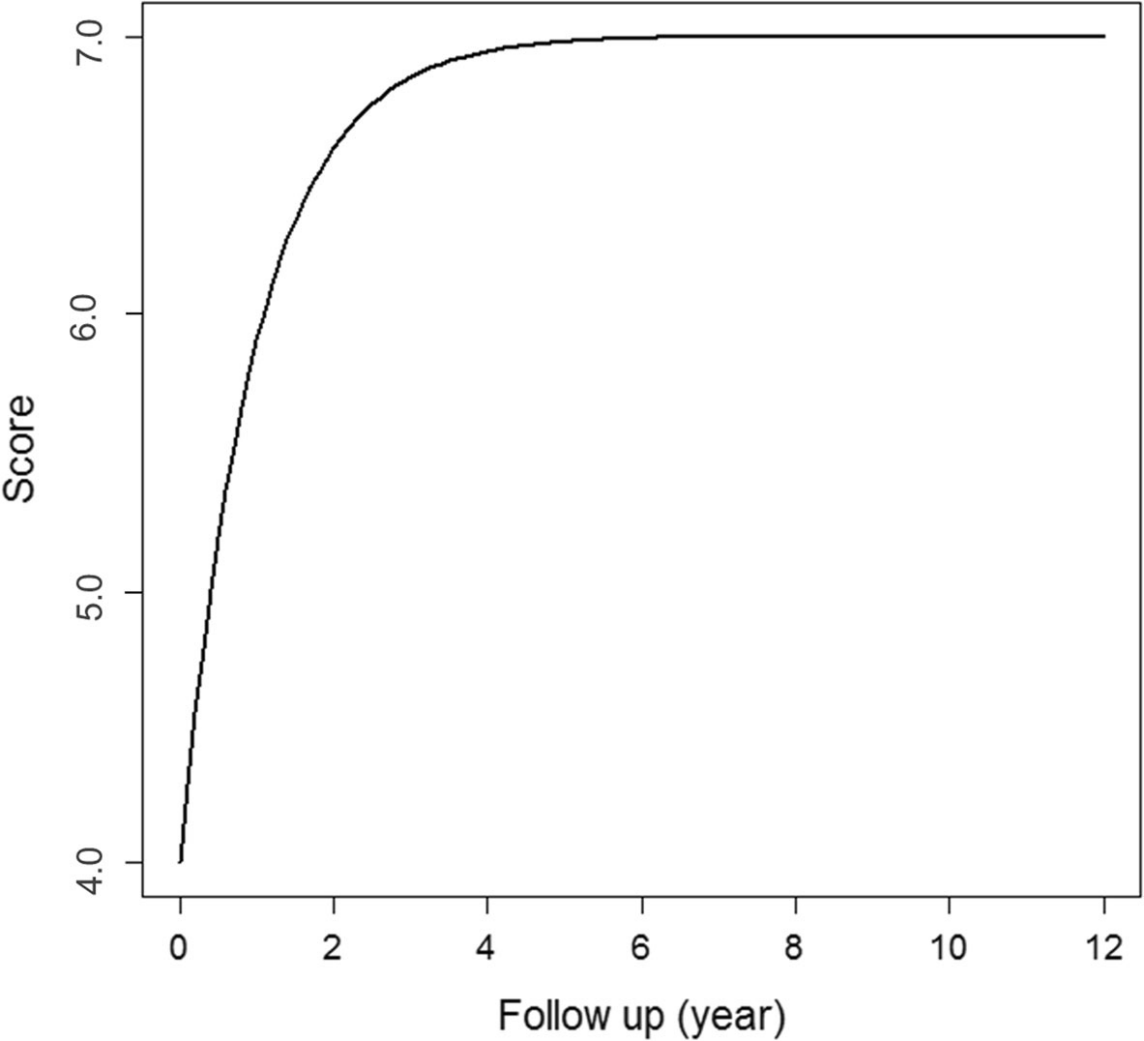
Graph showing the mean Goldberg score according to the duration of follow-up after Dega osteotomy

**Fig. 4:**
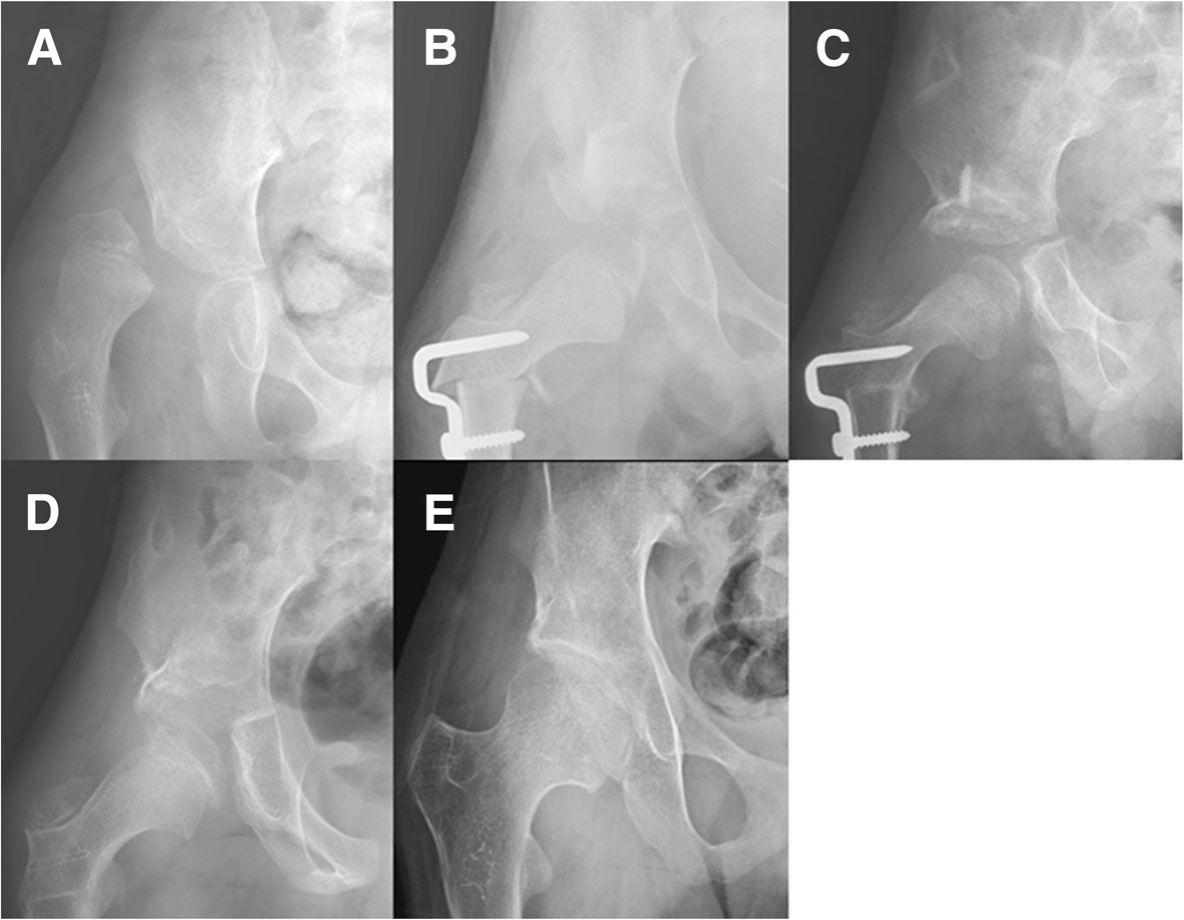
**a** Preoperative hip radiograph of a 9-year-old body with cerebral palsy shows right hip dislocation and aceteabular dysplasia. **b** He underwent hip reconstructive surgery including Dega osteotomy using iliac crest allograft for right hip. **c** The osteotomy site was not incorporated yet at 6 months after the surgery (Goldberg score of 5), **d** It was completely incorporated at 1 year after the surgery (Goldberg score of 7). **e** Correction of acetabular dysplasia remained stable at 10 years after the surgery

The mean estimated Goldberg score was 6 at 1.1 years after Dega osteotomy (Fig. 3). Twenty-four hips (16%, 4 hips with GMFCS level IV and 20 hips with GMFCS level V) were classified as radiographic delayed union (Goldberg score < 6) at 6 months after surgery. Nine hips (6%, all hips with GMFCS level V) had Goldberg score < 6 at 1 year after surgery. However, all hips showed radiographic union at the final follow-ups and no hips underwent reoperation due to nonunion. There were no cases of bone graft resorption, nonunion, dislodgement, and graft-related infections (Fig. 4).

GMFCS level was significantly associated with radiographic delayed union (*p* = 0.001). Patients with GMFCS level V had 6.9 times higher risks for radiographic delayed union than those with GMFCS level III and IV. Other factors such as age, sex, anatomical type and body side were not associated with radiographic delayed union (Table 4).

**Table 4:** Potential risk factors for radiographic delayed union

Factor	Adjusted OR (95% CI)	*P*-value
Age (per year)	0.9 (0.8 to 1.1)	0.443
Sex (male)	0.4 (0.2 to 1.0)	0.062
GMFCS level (V)	6.9 (2.2 to 22.2)	0.001
Anatomical type (quadriplegia)	2.3 (0.2 to 22.5)	0.476
Body side (right)	1.6 (0.6 to 4.0)	0.365

*OR* odds ratio, *CI* confidence interval, *GMFCS* Gross Motor Function Classification System; Multivariate analysis using generalized estimation equation is used to calculate the OR and CI

AI was not increased by follow-up duration (0.2 degrees per year; *p* = 0.316). However, MP and NSA were significantly increased by follow-up duration (2.5%, *p* < 0.001 and 2.5 degrees, *p* < 0.001, respectively) (Table 5).

**Table 5:** Factors affecting radiographic measurements after hip reconstructive surgery

	Acetabula index			Migration percentage			Neck-shaft angle		
	Estimate	SE	*P*-value	Estimate	SE	*P*-value	Estimate	SE	*P*-value
Follow-up duration (year)	0.2	0.2	0.316	2.5	0.2	< 0.001	2.5	0.3	< 0.001
Age at surgery	−0.0	0.2	0.919	0.2	0.2	0.349	−1.3	0.3	< 0.001
Sex	0.0	0.8	0.961	−2.3	1.1	0.036	2.5	1.7	0.127
GMFCS level									
V (reference)									
III	3.1	1.2	0.010	3.8	1.5	0.013	9.1	2.4	< 0.001
IV	1.2	0.9	0.198	0.6	1.2	0.600	3.1	1.8	0.086
Anatomical type	−0.2	1.2	0.873	−0.1	2.0	0.966	−2.2	2.8	0.445
Laterality	−1.1	0.8	0.001	−0.1	1.0	0.451	−1.2	1.6	0.835

A linear mixed model was used to estimate factors affecting AI, MP and NSA

*SE* standard error, *GMFCS* Gross Motor Function Classification System

## Discussion

To our knowledge, this is the largest study investigating outcomes after Dega osteotomy and the first study regarding the allograft behavior after Dega osteotomy in patients with CP. This study showed that a Dega pelvic osteotomy using an allograft could not only correct acetabular dysplasia, but also keep it stable over time. Therefore, an allograft can be a good option as the interposition material for Dega osteotomy if a femoral autograft is not available. Additionally, this study found that allograft incorporation in patients with GMFCS Level V was significantly delayed compared to those with GMFCS level III and IV.

There were some limitations of this study. First, only retrospective review of medical records and radiographic assessments were used for evaluating surgical outcomes. However, we believe that allograft behavior can be reflected best by radiographic assessment. Second, all patients were not evaluated until skeletal maturity. However, all hips showed radiographic union at final follow-up without any allograft-related complication. Furthermore, our analysis showed that the correction of acetabular dysplasia remained stable throughout the follow-up duration. Therefore, we think that further follow-up may not be necessary. Thirds, no comparison group that used autograft for Dega osteotomy was included. Therefore, further study comparing the outcomes of allografts and autografts as graft materials for Dega osteotomy is required.

Most of authors used the iliac crest autograft or femoral autograft obtained from femoral shortening osteotomy as a bone graft material for Dega osteotomy and showed good clinical and radiological outcomes in patients with CP and developmental dysplasia of the hip (DDH) (Table 6). [6, 29–45] Mallet et al. investigated the long-term results after one-stage hip reconstructive surgery in children with CP. [37] They found that correction of AI remained stable postoperatively for 9 years of follow-up. Jozwiak et al. also reported that AI did not show any noticeable changes during the follow-up period after Dega pelvic osteotomy in patients with CP. [31] Our study also showed that AI did not increase during the follow-up period.

**Table 6:** Previous studies on the outcome after Dega osteotomy

Author	Diagnosis	Graft material	No. of hips	Age at surgery (year)	Follow-up duration (year)	AI (°)			MP (%)			NSA (°)		
						Preop	Postop	final	Preop	Postop	final	Preop	Postop	final
Current study	CP	Iliac crest allograft	150	8.7	2.9	32.2	13.6	13.8	75.2	0.5	11.7	156	119.9	125.1
Mubarak [29]	CP	Iliac crest autograft	18	8.4	6.8	30		14	78		6.2	149		95
McNerney [30]	CP	Iliac crest autograft	104	8.1	6.9	26	13	11	66	5	14			
Jozwiak [31]	CP		30	7.0	12.0	32	22	23	65	11	20	152	133	140
Robb [32]	CP	Femoral autograft	52	14.0	4.0				70	10				
Kim [33]	CP	Iliac crest or femoral autograft	32	8.6	2.3	35.7	19		74.2	10.6				
Dhawale [34]	CP		22	7.5	11.7				79.4	4.3	7.9	151	112	120.6
Koch [35]	CP	Femoral autograft	115	9.0	5.5	30.7	21.3		98.3	16		142	119.6	119.3
Braatz [36]	CP	Femoral autograft		7.3	7.7				68	12	16			
Mallet [37]	CP	Femoral autograft	20	8.1	9.1	30.1	12.7	15.8	60.6	4.9	15.4	153	114.6	129.7
Reidy [38]	CP	Femoral autograft	57	8.9	5.4				63.6	2.7	9.7	152	132.6	137.2
Grudziak [39]	DDH	Iliac crest or femoral autograft or fibular allograft	24	5.8	4.6	33	12							
Karlen [40]	DDH	Iliac crest or femoral autograft	26	3.1	4.3	37	15	13						
	NM		24	6.3	4.7	36	16	14	84	8	14			
Wade [6]	DDH	Iliac crest allograft	147	2.9	2.0	43.2	24.3	16.9						
Al-Ghamdi [41]	DDH		21	4.6	7.3	37	17	19	38	−10	15			
Aksoy [42]	DDH	Iliac crest or femoral autograft	43	2.9	4.8	35	20	13						
Akgul [43]	DDH		26	3.2	3.5	39.4	18.3	15						
El-Sayed [44]	DDH	Iliac crest or femoral autograft	58	4.1	16.6	39	18	25		−21	19			
Issin [45]	DDH	Iliac crest autograft	10	2.1	5.6	46	23.4	15.9						

*CP* cerebral palsy, *DDH* developmental dislocation of hip, *NM* neuromuscular, *AI* acetabular index, *MP* migration percentage, *NSA* neck-shaft angle

On the contrary, previous studies have found that both NSA and MP showed a tendency to worsen during the follow-up period after hip reconstruction, including Dega osteotomy, in CP . [31, 37] In addition, Bayusentono et al. showed that MP significantly increased by 2.0% per year in patients with GMFCS level IV and by 3.5% per year in those with GMFCS level V. [24] Our study also showed that MP and NSA were significantly increased during the follow-up period, as reported in previous studies.

Several studies showed good surgical outcome after pelvic osteotomy using allograft for DDH patients. Wade et al. investigated the radiologic results of 147 hips treated for DDH by Dega osteotomy with an iliac crest allograft. [6] They showed that postoperative corrected AI had improved at 2 years of follow-up. McCarthy et al. compared the results of autograft and allograft in 36 hips after Pemberton osteotomy. [46] Almost all of the children with DDH had satisfactory results regardless of graft type, but allograft provided better results than iliac crest autograft in neuromuscular diseases. Kessler et al. also reported that allograft bone could be effectively used in Pemberton osteotomy in 26 hips with DDH or neuromuscular disorders. [47] The authors believed that the immediate stability, owing to the larger size and the mechanical properties of the graft, allowed for earlier rehabilitation.

Patients with CP have low BMD, which is highly correlated with GMFCS levels. Several factors, including physical disability, poor nutritional status, decreased calcium intake, low vitamin D level, prolonged immobilization, sarcopenia, and the use of anticonvulsant, were associated with the low BMD in patients with CP. [48–50] Moon et al. showed that bone attenuation of the acetabulum and femur neck was significantly affected by GMFCS levels and degree of hip displacement. [7] Because the osteoporotic features around hip joints in CP may not guarantee the initial mechanical stability of osteotomy site, we had used iliac crest allograft as the interposition material at the osteotomy site.

Allograft has been proven to be a good choice of graft in other pediatric orthopedic conditions. Wade et al. showed that all of the allografts were completely incorporated at 6 months after surgery with a mean incorporation time of 3 months in 147 hips treated for DDH by Dega osteotomy. [6] Lee et al. investigated the incidence and risk factors of allograft failure after lateral column lengthening for planovalgus foot deformity. [19] They reported that the mean estimated Goldberg score was 6 at 6 months after surgery and 4% of feet had Goldberg score < 6 at 6 months after surgery. Additionally, reoperation using an autogenous iliac bone graft bone was performed in four feet (1%). In our study, the mean estimated Goldberg score was 6 at 1.1 years after Dega osteotomy and at 6 months after the surgeries, 16% of hips had a Goldberg score of < 6. Furthermore, allograft incorporation in patients with GMFCS Level V was significantly delayed than in those with GMFCS level III and IV. However, no hip underwent reoperation due to allograft failure. We think that the delayed allograft incorporation in our study, compared with previous studies, is due to the underlying CP in the included patients with GMFCS level III to V. On the other hand, Wade et al.’s study included patients with DDH, and Lee et al.’s study included patients with idiopathic planovalgus and ambulatory CP. We think that the delayed allograft incorporation in patients with GMFCS level V compared with those with GMFCS level III and IV is due to the severity of osteoporosis. Therefore, surgeons should remember that the degree of osteoporosis might affect the time to allograft incorporation and pay extra attention to the patients with GMFCS level V.

## Conclusion

Dega pelvic osteotomy using iliac crest allograft was an effective procedure in the correction of acetabular dysplasia without graft-related complications in patients with CP. Additionally, the correction of acetabular dysplasia remained stable during the follow-up period. However, physicians should consider that allograft incorporation in patients with GMFCS level V can be delayed compared with those with GMFCS level III & IV.

## Abbreviations

AI: Acetabular index
CP: Cerebral palsy
DDH: Developmental dysplasia of hip
FVO: Femoral varization osteotomy
GEE: Generalized estimating equation
GMFCS: Gross Motor Function Classification System
LMM: Linear mixed model
MP: Migration percentage
NSA: Neck-shaft angle
